# Human Mesenchymal Stem Cells Seeded on the Natural Membrane to Neurospheres for Cholinergic-like Neurons

**DOI:** 10.3390/membranes11080598

**Published:** 2021-08-07

**Authors:** Priscila Elias Ferreira Stricker, Daiany de Souza Dobuchak, Ana Carolina Irioda, Bassam Felipe Mogharbel, Celia Regina Cavichiolo Franco, José Roberto de Souza Almeida Leite, Alyne Rodrigues de Araújo, Felipe Azevedo Borges, Rondinelli Donizetti Herculano, Carlos Frederico de Oliveira Graeff, Juan Carlos Chachques, Katherine Athayde Teixeira de Carvalho

**Affiliations:** 1 Advanced Therapy and Cellular Biotechnology in Regenerative Medicine Department, Child and Adolescent Health Research and Pequeno Príncipe Faculties, Pelé Pequeno Príncipe Institute, Curitiba 80240-020, Brazil; priscilaeferreira@gmail.com (P.E.F.S.); daianys.bio@gmail.com (D.d.S.D.); anairioda@gmail.com (A.C.I.); bassamfm@gmail.com (B.F.M.); 2 Cell Biology Department, Federal University of Paraná, Curitiba 81530-000, Brazil; crcfranc@terra.com.br; 3 Research Center in Applied Morphology and Immunology, NuPMIA, Faculty of Medicine, University of Brasília, Brasília 70910-900, Brazil; jrsaleite@gmail.com; 4 Biodiversity and Biotechnology Research, Parnaíba Delta Federal University, Parnaíba 64202-020, Brazil; alyne_biomed@hotmail.com; 5 Faculty of Pharmaceutics Sciences, São Paulo State University (UNESP), Araraquara 14800-903, Brazil; felipeazevedoborges@hotmail.com (F.A.B.); rond.donizetti@gmail.com (R.D.H.); 6 Physics Department, São Paulo State University (UNESP), Bauru 17033-360, Brazil; carlos.graeff@unesp.br; 7 Laboratory Biosurgical Research, Cardiovascular Division, Pompidou Hospital, University of Paris, 75015 Paris, France; j.chachques-ext@aphp.fr

**Keywords:** mesenchymal stem cells, umbilical cord, blood, membrane, biopolymer, cholinergic neurons

## Abstract

This study aimed to differentiate human mesenchymal stem cells (hMSCs) from the human umbilical cord in cholinergic-like neurons using a natural membrane. The isolation of hMSCs from Wharton’s jelly (WJ) was carried out using “explant” and mononuclear cells by the density gradient from umbilical blood and characterized by flow cytometry. hMSCs were seeded in a natural functional biopolymer membrane to produce neurospheres. RT-PCR was performed on hMSCs and neurospheres derived from the umbilical cord. Neural precursor cells were subjected to a standard cholinergic-like neuron differentiation protocol. Dissociated neurospheres, neural precursor cells, and cholinergic-like neurons were characterized by immunocytochemistry. hMSCs were CD73+, CD90+, CD105+, CD34- and CD45- and demonstrated the trilineage differentiation. Neurospheres and their isolated cells were nestin-positive and expressed NESTIN, MAP2, ßIII-TUBULIN, GFAP genes. Neural precursor cells that were differentiated in cholinergic-like neurons expressed ßIII-TUBULIN protein and choline acetyltransferase enzyme. hMSCs seeded on the natural membrane can differentiate into neurospheres, obtaining neural precursor cells without growth factors or gene transfection before cholinergic phenotype differentiation.

## 1. Introduction

The World Health Organization (WHO) has included Alzheimer’s Disease (AD) among the major health problems, making it essential to develop diagnostic strategies and treatments for this disease. According to the WHO, about 35.6 million people had the disease in 2010, and this number might reach 65.7 million in 2030 and 115.4 million in 2050. According to a report issued in 2012 by the WHO, and The International Association for Alzheimer’s Disease, the number of people with dementia in the world can be compared to the spread of HIV/AIDS in the 1980s. The report presents this comparison to warn the world about the importance of alternative treatments for AD [1,2].

In this context, cell therapies have become one of the research targets that may contribute to the treatment of neurodegenerative diseases, such as AD. Among cell types, the mesenchymal stem cells (MSC) emerge as an option of cells that are easy to obtain and present pluripotency such as cholinergic-like neurons to prospect functional repair of nervous tissue. It seems relevant to emphasize that MSC could be easily obtained from adipose tissue and from the umbilical cord, both discharged materials that require the approval of ethics committees.

On the other hand, to obtain neural precursor cells (including neural stem cells and neural progenitor cells), which rise to neurons, it is necessary to establish protocols for developed neurospheres [3]. When the neurospheres are obtained from mesenchymal stem cells, the authors need to use various growth factors and/or gene transfection for the induction of neural precursor cells. According to Mukai et al., 2016, several neural differentiation protocols are reported, and the culture of neurospheres is widely described in the literature because it is a crucial way to expand the production of neural stem cells (NSCs). The vast majority of articles use the formation of neurospheres for further neural differentiation, or preclinical application. The differentiations performed are basically for oligodendrocytes, glial cells, and neural cells in general [4,5,6,7,8].

The need for adhesive properties for cell anchorage of the mesenchymal stem cells requires interaction with the culture flask substrate in the cell culture as well as the membrane with the cells in the tissue, in vivo; in both conditions there are cell–membrane relations.

**Scheme 1:**
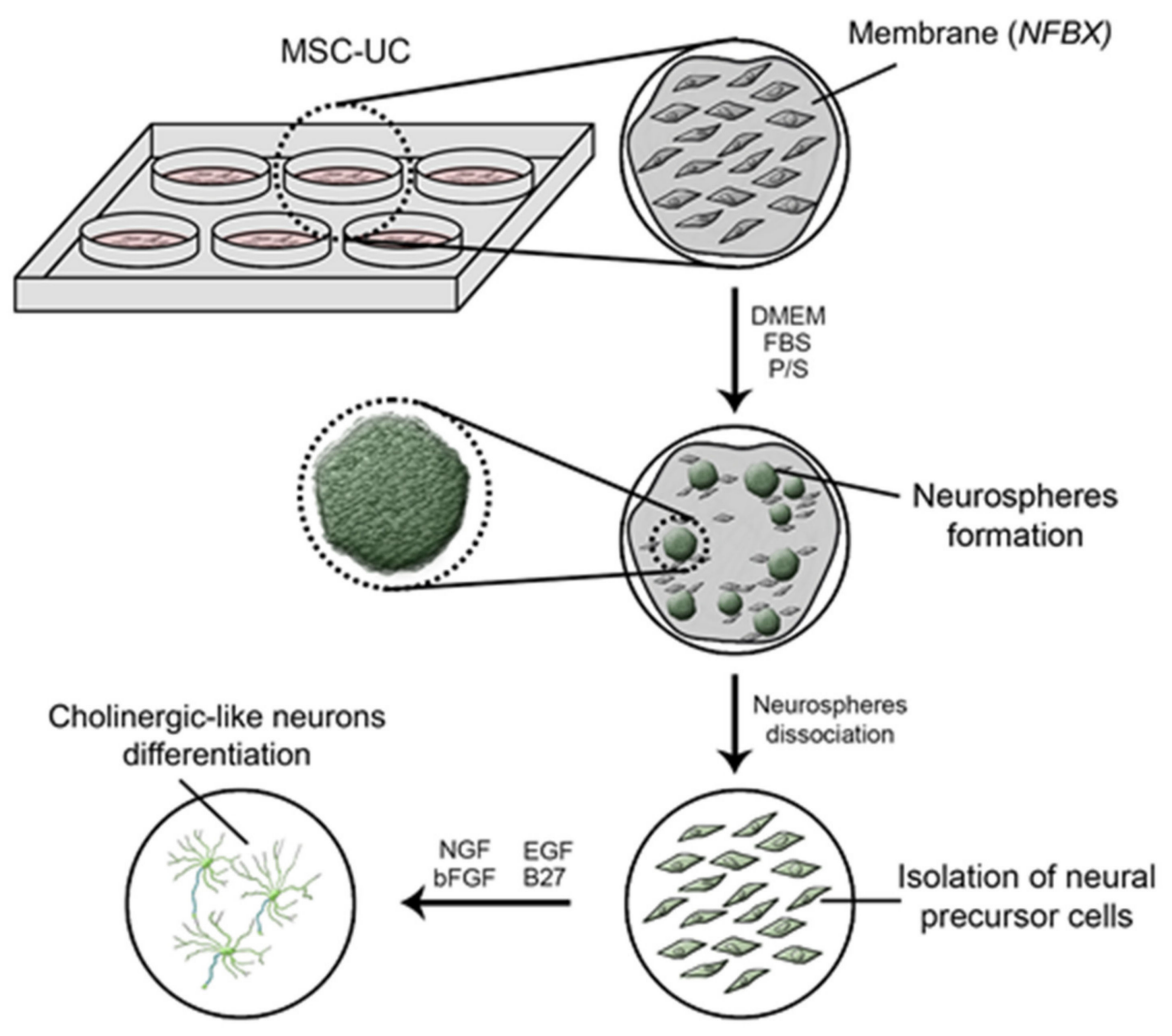
Neurospheres until cholinergic-neurons differentiation. The MSC-UC were seeded on in NFBX to obtained neurospheres. Neurospheres were than mechanical dissociated to obtain the through the neurospheres the neural precursor cells. The neural precursor cells were induced to cholinergic-like neurons through the addition of NGF, bFGF, EGF, and B27 in cultivation media. Abbreviations: MSC-UC: Umbilical cord mesenchymal stem cells; NFBX: Natural Functional Biopolymer Matrix; DMEM: Dulbecco’s Modified Eagle Medium; FBS: fetal bovine serum; P/S: penicillin and streptomycin, NGF: nerve growth factor; EGF: epidermal growth factor, bFGF: basic fibroblast growth factor, B27: neuronal supplementation.

The matrices could act as epigenetic factors for stemness of cell fate to select the cellular type of cells, not necessarily acting only as scaffold. In this study, a natural functional biopolymer matrix named NFBX as a membrane was used. Its characteristics allow those aggregated MSCs to express grouped nestin-positive cells, like spheres. After that, the spheres were demonstrated to have most nestin-positive cells. Nestin is a protein present in neural precursor cells. Some description of this membrane is presented in the material and methods. Scheme 1 showed the demonstration of neurospheres until cholinergic-neurons differentiation.

This research project aims to evaluate the possibility of differentiation of MSCs from the human umbilical cord in nestin-positive neural precursor cells (NPCN+) through the NFBX into cholinergic “like” neurons. Following the establishment of the differentiation protocol, NPCN+ and cholinergic-like neurons derived from the mesenchymal stem cells derived from Wharton jelly (MSC-WJ) and umbilical cord blood (MSC-UCB), could be evaluated in preclinical models of neurodegenerative disease therapy, for example in AD [9,10,11].

## 2. Materials and Methods

### 2.1. Umbilical Cord Stem Cells-Ethical Donors

The Research Ethics Committee of Pequeno Príncipe Faculty approved this study, which was numbered 1.320.984. (16 November 2015). Then, the human umbilical cords (HUCs) were collected after the donor/responsible person had signed the informed consent form. Donor/responsible people do not have the identities revealed, and all methods were performed with the agreement of the Research Ethics Committee of Pequeno Príncipe Faculty. The collections were completed at term, shortly after the placenta was discharged. Four HUC samples were collected and used in this study in triplicate for stem cell isolation. Two of these cords were also used for both Wharton jelly and human umbilical cord blood mesenchymal stem cell isolation.

### 2.2. Isolation of Stem Cells from Wharton Jelly

Explant Method: HUCs were washed with phosphate buffer saline (PBS) with 3% antibiotic (300 IU/mL penicillin, 0.3 mg/mL streptomycin), the longitudinal intersection was performed, and the vein and arteries were removed. The tissue was fragmented into 2 mm × 2 mm pieces, which were seeded onto culture plates containing DMEM/Ham-F12 (Sigma Aldrich, St. Louis, MO, USA) supplemented with 10% Fetal bovine serum (FBS) and 1% antibiotic (100 IU/mL penicillin, 0.1 mg/mL streptomycin) and incubated at 37 °C and 5% CO_2_. The new replacement of the medium was performed after five days of culture. After that, the medium was replaced every 72 h up to 85% of confluence [12].

### 2.3. Isolation of Stem Cells from Human Umbilical Cord Blood

Density gradient method: human umbilical cord blood was collected through the open syringe system containing heparin and processed within 24 h. Mononuclear cells were isolated using the Ficoll-Paque PLUS^®^ (GE Healthcare Life Sciences, Chicago, IL, USA) density gradient method. In this method, three parts of blood were added to one part of Ficoll-Paque PLUS^®^ in the tube; then, the tube was centrifuged at 22 °C, 1200 rpm for 30 min, after that, the phase containing the mononuclear cells was removed. Then these cells were washed in PBS containing 2% FBS at 1:1 ratio and centrifuged at 1200 rpm for 10 min. The pellet cells were resuspended in 4 mL DMEM/Ham-F12 culture medium with 20% FBS. Subsequently, 1 × 10^7^ cells/mL were seeded in 25 cm^2^ culture flasks and incubated at 37 °C and 5% of CO_2_ [13,14].

### 2.4. Mesenchymal Stem Cells Characterization

The characterization of MSCs was performed according to the International Society of Cell Therapy. For the immunophenotypic characterization of MSCs, the markers used were CD105, CD73, CD90, CD29, CD34, CD45, and 7-AAD, the last one was used for cell viability. Cells were subjected to trypsinization and suspended in PBS at the concentration of 1 × 10^6^ cells/mL. From this suspension, 200 μL were placed in correctly identified tubes, and corresponding antibodies were added and incubated for 20 min. After that, 400 μL of PBS was added to the tubes and centrifuged for 5 min/1200 rpm. The supernatant was discarded, and the pellet suspended in 400 μL PBS, followed by homogenization and 10 μL 7-AAD added. Finally, the samples were processed in a flow cytometer (FACS Calibur; Becton Dickinson, Franklin Lakes, NJ, USA) and analyzed using Infinicyt Flow Cytometry software, Version 1.6.0 (Histograms are shown in Figure S1, [15]. The gating strategy was carried out excluding non-viable cells (positive for the 7-AAD marker) and comparing each CD marker with isotype control. Thus, all marking that overlapped the isotypic control was considered negative for the analyzed marker, any marking that did not overlap the isotypic control, or to its left, was considered positive for the analyzed marker.

To verify the multipotent characteristic of the isolated MSCs, adipogenic, osteogenic, and chondrogenic differentiation were performed. Commercial kits were used in these differentiations: StemPro^®^ Adipogenesis Differentiation Kit, StemPro^®^ Osteogenesis Differentiation Kit, and StemPro^®^ Chondrogenesis Differentiation Kit, respectively. After differentiation, the samples were subjected to their specific staining: osteogenic—Alizarin Red—Oil Red O and chondrogenic—Alcian Blue (Sigma Aldrich, St. Louis, MO, USA).

### 2.5. Preparation of the Polyisoprene-Based Membrane

To prepare the membrane, it was based on polyisoprene (C5H8), which is the primary chemical constituent of natural rubber extracted from *Hevea brasiliensis*. The method of preparation of the polyisoprene-based membrane is from the latex (COLITEX^®^, São Paulo, Brazil) in dilution (*v*/*v*), 1:2 in aqueous solution, pure water followed by exposure to ultraviolet rays of Laminar Flux for overnight sterilization. Then, the cellular culture flasks were coated, in the proportion of 0.5 mL/cm^2^ on the polystyrene surface of the cellular plate, on the side where cell culture occurs. The polyisoprene polymerizes at ambient temperature after 12 h and is ready for use: cells seedings. Forward, this polyisoprene-based membrane was named natural functional biopolymer matrix (NFBX).

### 2.6. Production of Neural Precursor Cells

To produce neural precursor cells, was used the NFBX as a membrane. This membrane was coated on the culture flasks and developed the neurospheres when a density plating of 2 × 10^4^ hMSCs/mL and DMEM/Ham-F12, 100 UI/mL penicillin, 100 μg/mL streptomycin culture medium with 20% FBS incubated at 37 °C and 5% of CO_2_. A period for the neurospheres appears, according to the sample, around 15 to 21 days, as it is a primary culture. Cell cultures are observed daily. Then, the neurospheres transferred with tweezers to other flasks without membrane, and neural precursor cells migrated from the neurospheres. The neurospheres and neural precursor cells were both nestin-positive, as demonstrated by immunocytochemistry methods. This method was schematized in the graphical abstract.

The NFBX is briefly explained to understand of the interaction of hMSCs with this membrane on their morphology that permits the attachment of the selection of cells committed to being neural precursors. These facts require further studies on the mechanisms of mechanic-physicochemical processes to determine the geometry of the attached neurospheres.

### 2.7. Cholinergic-like Neurons Differentiation Protocol

After the neurospheres production, the culture medium (DMEM/Ham-F12, 100 UI/mL penicillin; 100 μg/mL streptomycin) was supplemented with nerve growth factor (NGF-Peprotech^®^, USA), epidermal growth factor (EGF-Peprotech^®^, East Windsor, NJ, USA), basic fibroblast growth factor (bFGF-Peprotech^®^, East Windsor, NJ, USA) and B27 (Gibco^®^ BRL, Life Technologies, Inc., Grand Island, NY, USA) for 11 days (Table S1). The final concentration was maintained for four days, after which phenotypic characterization tests were performed [14].

### 2.8. Immunocytochesmistry

The cells were fixed with 4% paraformaldehyde (Sigma Aldrich, St. Louis, MO, USA) for 20 min, washed with PBS and made permeable with a PBS solution containing 3% Triton X-100 (Sigma Aldrich, St. Louis, MO, USA) and 1% BSA for 5 min at room temperature, being washed three times with PBS after this period. The cells were then incubated overnight at 4 °C with the primary antibodies. The primary antibody used to characterize NPCN + was the anti-nestin monoclonal antibody (Sigma Aldrich, St. Louis, MO, USA). After differentiation in cholinergic “like” neurons, the primary antibody used was anti-ßIII-TUBULIN (Sigma Aldrich, St. Louis, MO, USA) and anti-ChAT (Merck Millipore, Burlington, MA, USA).

After 24 h incubated with the primary antibodies, the cells were washed with PBS and incubated at room temperature with the secondary antibody FITC-IgG (Sigma Aldrich, St. Louis, MO, USA). The undifferentiated control was obtained using MSC cultured only with standard medium (DMEM/Ham-F12, 100 UI/mL penicillin: 100 μg/mL streptomycin and 10% FBS). The reading was performed in an inverted fluorescence microscope (Axio Vert A1, Carl Zeiss, Oberkochen, Germany) [16,17].

### 2.9. Scanning Electron Microscopy

For the scanning electron microscopy (SEM) analysis, the MSC-WG was differentiated in neurospheres and cholinergic-like neurons. Then, the cells were washed with 0.1 M sodium cacodylate solution (PH 7.2 to 7.4), followed by fixation in Karnovski solution for 1 h and a half, dehydrated using alcohol solution 30%, 50%, 70%, 90%, 100%. The samples were subjected to the critical point (CPD-Balzers union, Baltec, Pfäffikon, Switzerland) and metalized with gold (CPD- Balzers union, Baltec, Pfäffikon, Switzerland). Then, cells were observed under scanning electron microscope (Vega-3LMU, Tescan, Brno, Czech Republic).

### 2.10. Qualitative Reverse Transcription-Polymerase Chain Reaction (RT-PCR)

The RNA was extracted using the RNeasy Mini Kit (Quiagen^®^), as indicated by the manufacturer. The complementary strand of DNA (cDNA) was generated precisely following the manufacturer’s instructions, using the High-Capacity cDNA (Thermo Fisher) kit. The RT-PCR was performed using a 20 µL system, containing the cDNA pattern and Master Mix Promega^®^. The primers used to amplify MAP2 (forward: CATACAGGGAGGATGAAGAGGG and reverse: GGTGGAGAAGGAGGCAGATTAG), GFAP (forward: ATCAGCCGATGCGAAGGG and reverse: TAGACGCTGATCCGCTCCAG), ßIII-TUBULIN (forward: ATTGAGTCGCTGGAGGAGGAGA and reverse: GGTAGTCGTTGGCTTCGTGCTT) and NESTIN (forward: AACAGCGACGGAGGTCTCTA and reverse: TTCTCTTGTCCCGCAGACTT) genes (SIGMA^®^). The samples were prepared in biological replicates, using ß-ACTIN control (forward: CTGGGACGACATGGAGAAAA and reverse: AAGGAAGGCTGGAAGAGTGC). Agarose gel was used, and the results were visualized on an ultraviolet transilluminator.

### 2.11. Atomic Force Microscopy Analysis

Morphological analysis of the membrane was carried out using a TT-AFM instrument (AFM Workshop, Billerica, MA, USA) in vibrating (tapping) mode with 512 × 512 lines. Representative images were examined using ACT-20 cantilevers (AppNano- Mountain View, CA, USA) with a resonant frequency of approximately 353 kHz. Images were analyzed using Gwyddion software 2.4, and the roughness value (Ra) for multiple areas of 1.5 × 1.5 µm (*n* = 15) examined is shown, Ra was expressed as mean ± SD.

## 3. Results

### 3.1. Flow Cytometry Analysis

In the flow cytometry test for MSC-WJ, an average of 91.27% of the mesenchymal stem cell characteristics was obtained, demonstrated by the expression for CD73, CD90, and CD105 markers. Out of these cells, 17.65% were 7AAD positive cells, i.e., non-viable cells. For the MSC-UCB derived sample, an average of 91.30% of the mesenchymal stem cell characteristics and 10.43% of non-viable cells were obtained and labeled 7AAD. When hematopoietic markers (CD34 and CD45) were considered, no expressive marking was observed for either of them, MSC-WJ or MSC-UCB. The gating strategy was achieved by excluding the cells that were marked 7-AAD (non-viable cells) and comparing the marking of each antibody with the isotype control. For the histograms and average of each protein expression, see Figure S1 in Supplementary Materials.

### 3.2. Trilineage Differentiation Test

**Figure 1:**
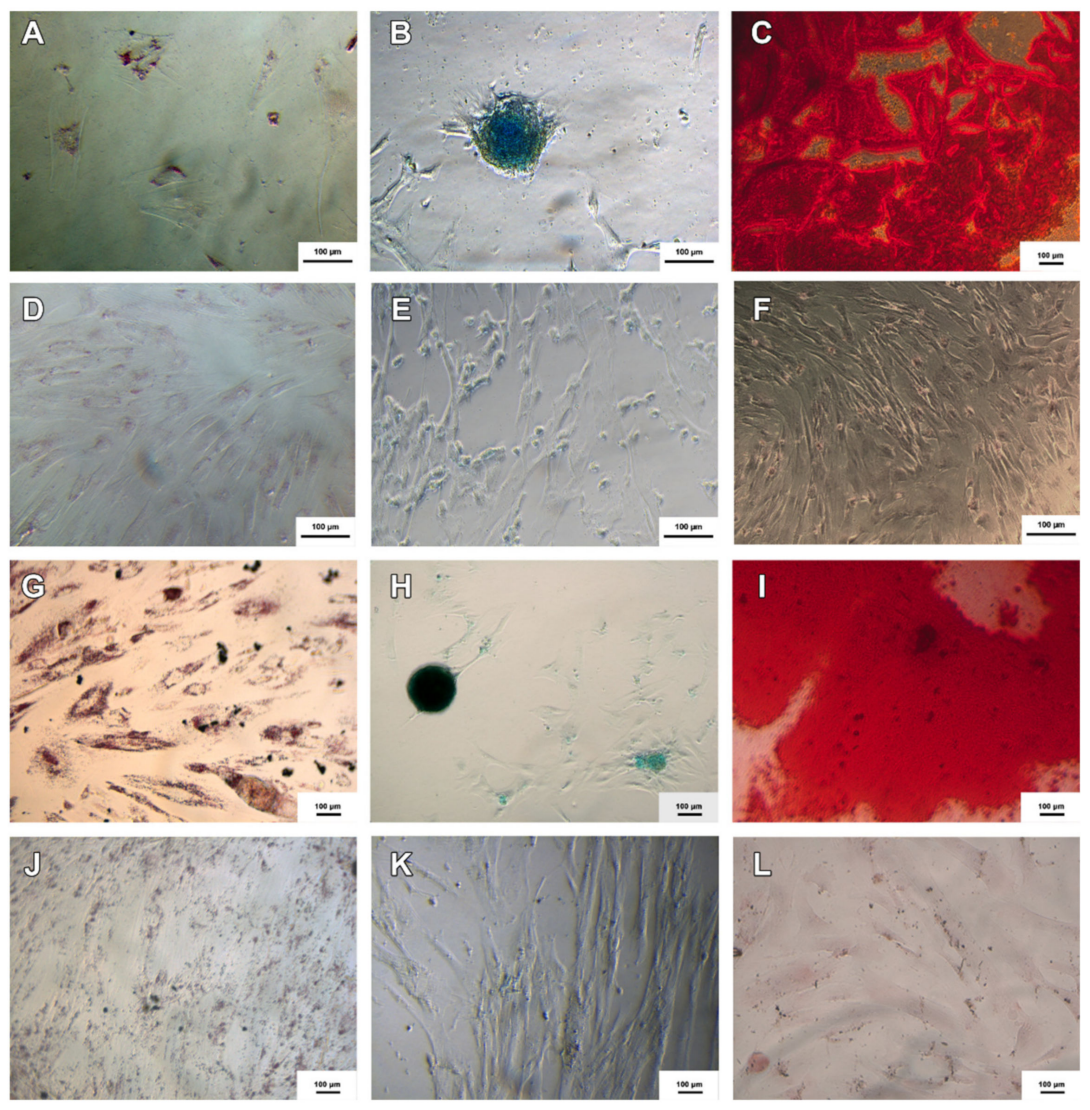
Adipogenic, chondrogenic and osteogenic differentiations of MSC-WJ and MSC-UCB. (**A**–**C**) represent the adipogenic, chondrogenic and osteogenic diferentiations of MSC-WJ respectively; And (**D**–**F**) represent the negative controls, respectively; (**G**–**I**) represent the adipogenic, chondrogenic and ostegenic differentiations of MSC-UCB respectively; And (**J**–**L**) the negative controls, respectively. Adipogenic, chondrogenic and osteogenic differentiations were stained with Oil Red O, Alcian Blue and Alizarin Red respectively (Inversion optical microscope, ×100).

Samples of MSC-WJ and MSC-UCB were successfully differentiated in adipogenic, chondrogenic, and osteogenic cells, known as trilineage differentiation test. A control test was performed using MSCs cultured only with standard medium (DMEM/Ham-F12, 100 UI/mL penicillin; 100 μg/mL streptomycin and 10% FBS) without differentiation stimulation, and these controls did not show specific staining (Figure 1).

### 3.3. Production of Neural Precursor Cells

**Figure 2:**
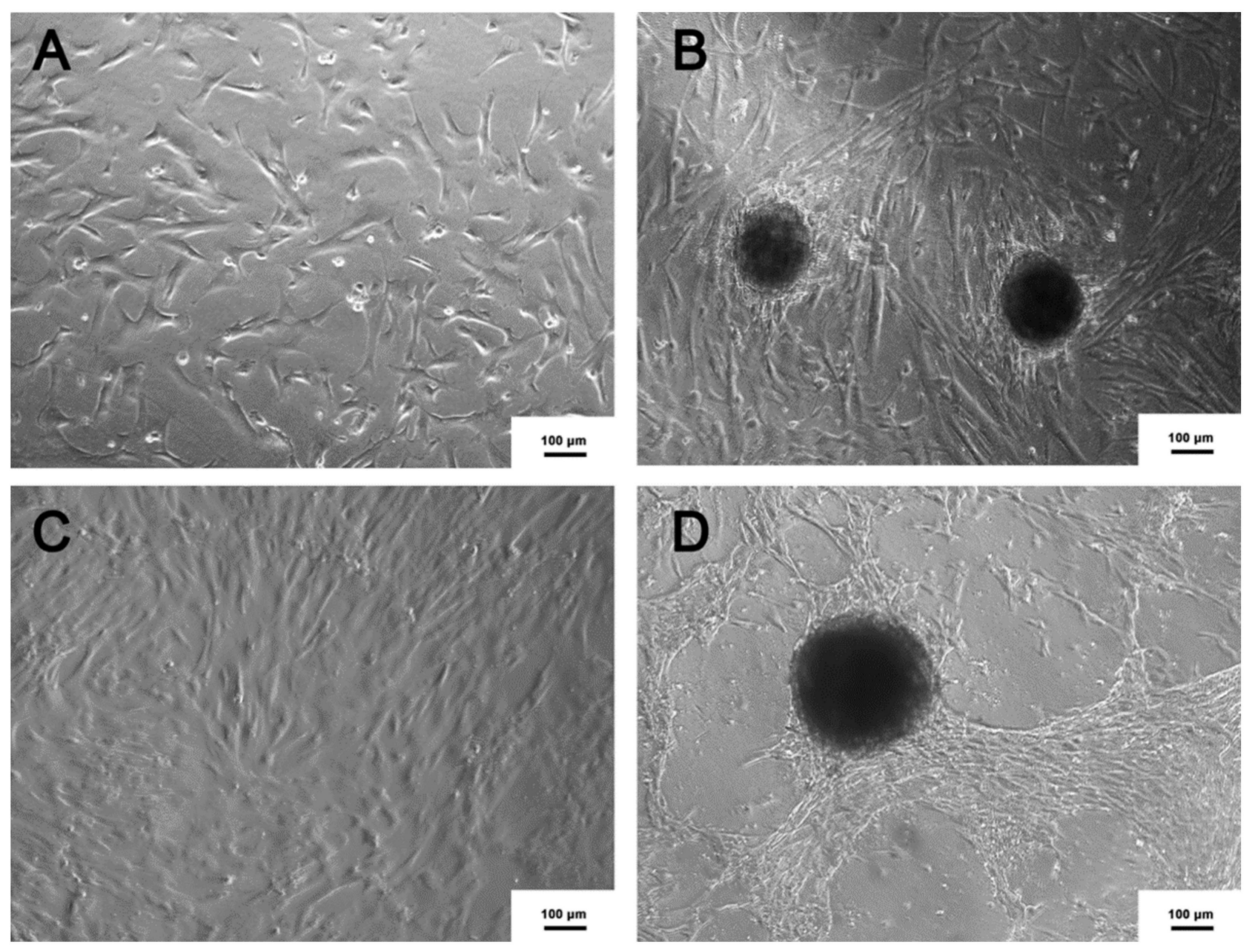
Neurospheres (NPCN+). (**A**) MSC-UCB seeded on polystyrene substrate (**B**) Neurospheres derived from MSC-UCB, seeded on NFBX. (**C**) MSC-WJ seeded on polystyrene substrate (**D**) Neurospheres derived from MSC-WJ, seeded on NFBX (Inversion optical microscope, ×100).

After two weeks of MSC-WJ seeded on NFBX, the formation of the neurospheres could be observed. On the other hand, the formation of neurospheres from the MSC-UCB cells could be observed only after a longer period, about three to four weeks, as shown in Figure 2.

### 3.4. Neurospheres and Neural Precursor Cells

**Figure 3:**
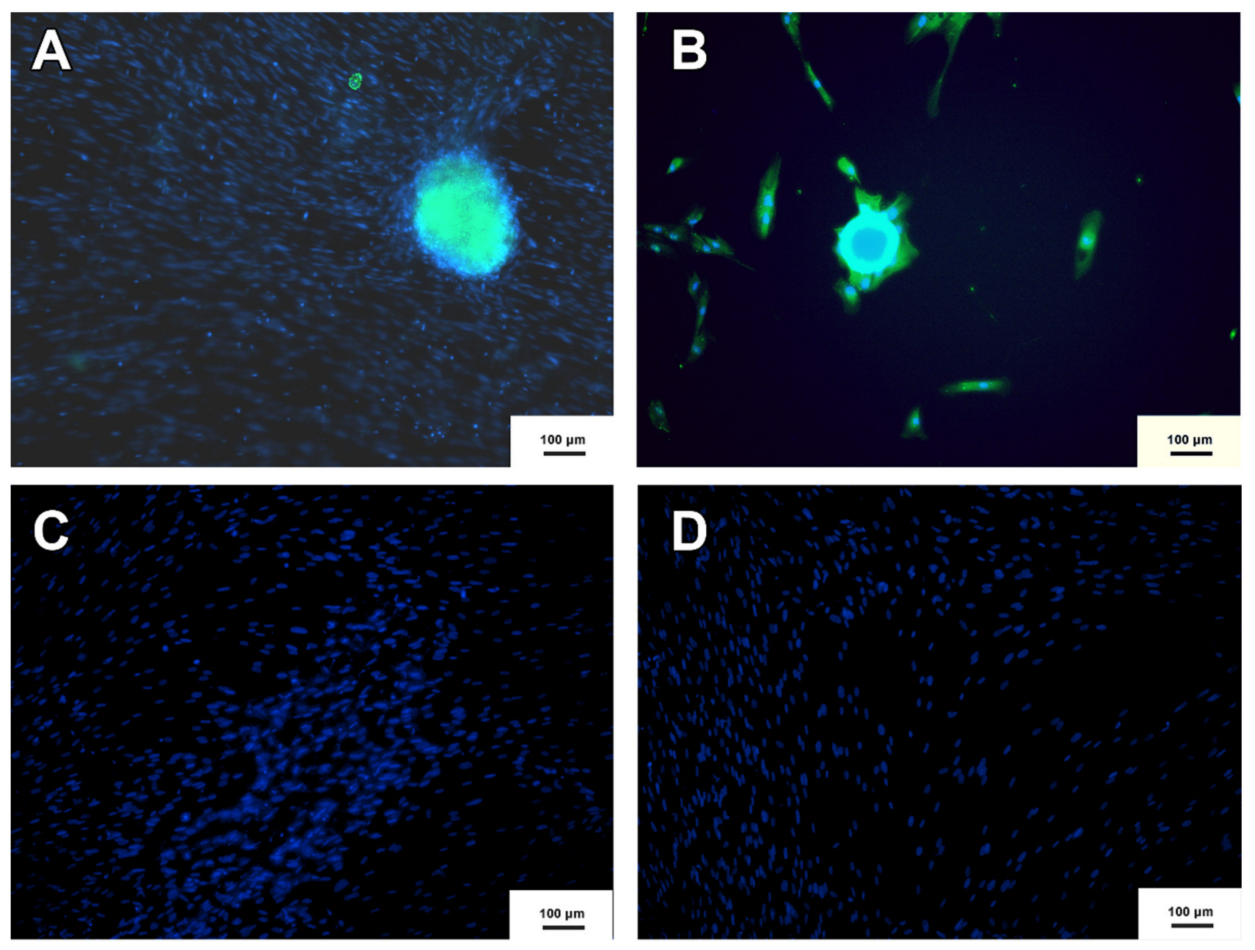
Neurosphere immunocytochemistry. (**A**) Neurosphere derived from MSC-WJ (**B**) Neurosphere derived from MSC-UCB. (**C**,**D**) represent the negative controls of both MSC-WJ and MSC-UCB respectively. This means that neither MSC-WJ nor MSC-UCB presented Nestin protein before being seeded in the NFBX. NESTIN protein is shown in green by FITC as a secondary antibody, and Hoechst was used for labeling the nuclei of the cells represented by blue (inverted fluorescence microscope ×100 (inverted fluorescence microscope Axio Vert A1, Carl Zeiss, Oberkochen, Germany).

The neurospheres produced were subjected to the immunocytochemistry protocol to verify the presence of the nestin protein. Nestin is considered a marker of neural precursor cells [18]. As shown in Figure 3, the neurospheres produced from MSC-WJ and MSC-UCB presented nestin expression and, therefore, were characterized as nestin-positive neural precursor cells.

### 3.5. Scanning Electron Microscopy

**Figure 4:**
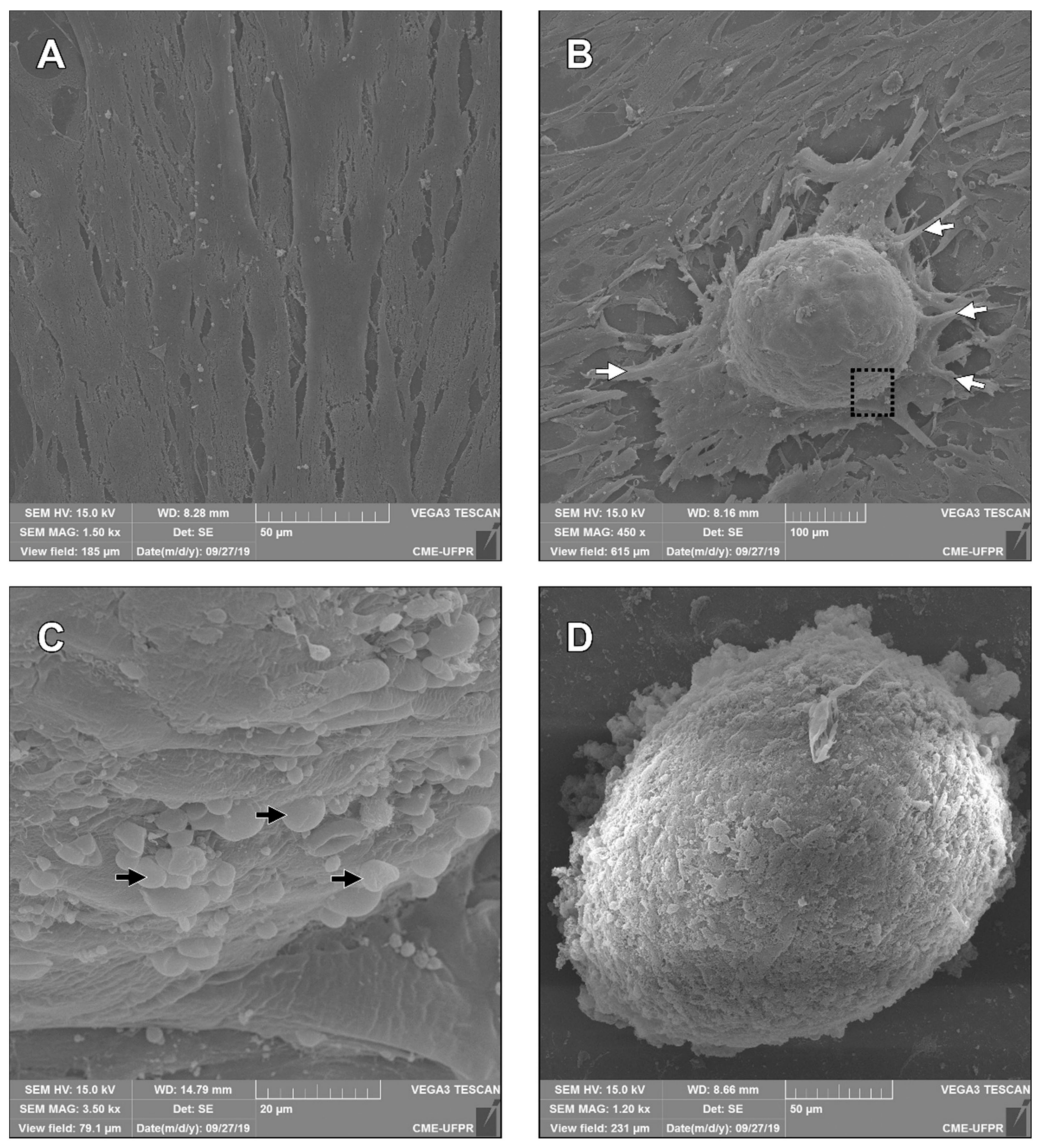
Neurospheres by scanning electron microscopy. (**A**) MSC-WJ reached confluency; (**B**) Neurospheres began to be formed by the MSC-WJ, these cells-initiated pseudopod projections for grouping and anchoring and began spherical organization (white arrows); (**C**) Cells that migrated and took part in the neurospheres formation (black arrows); (**D**) neurospheres formed by the MSC-WJ organization. SEM: Vega-3LMU, Tescan, Brno, Czech Republic.

The neural precursor cell from MSC-WJ and MSC-WJ neurospheres was observed using Scanning Electron Microscopy (SEM) (Vega-3LMU, Tescan, Brno, Czech Republic), presented in Figure 4. As shown in Figure 4A, MSC-WJ starts growing, reaches the confluency, and becomes the organizer in neurospheres (Figure 4B). Anchor cells could be observed in Figure 4B casting projections that are grouping structures. Finally, Figure 4C shown the cells that are grouped and participate in the neurospheres formation, and (Figure 4D) the unique neurosphere formed could be observed.

### 3.6. Qualitative Reverse Transcription–Polymerase Chain Reaction (RT-PCR)

**Figure 5:**
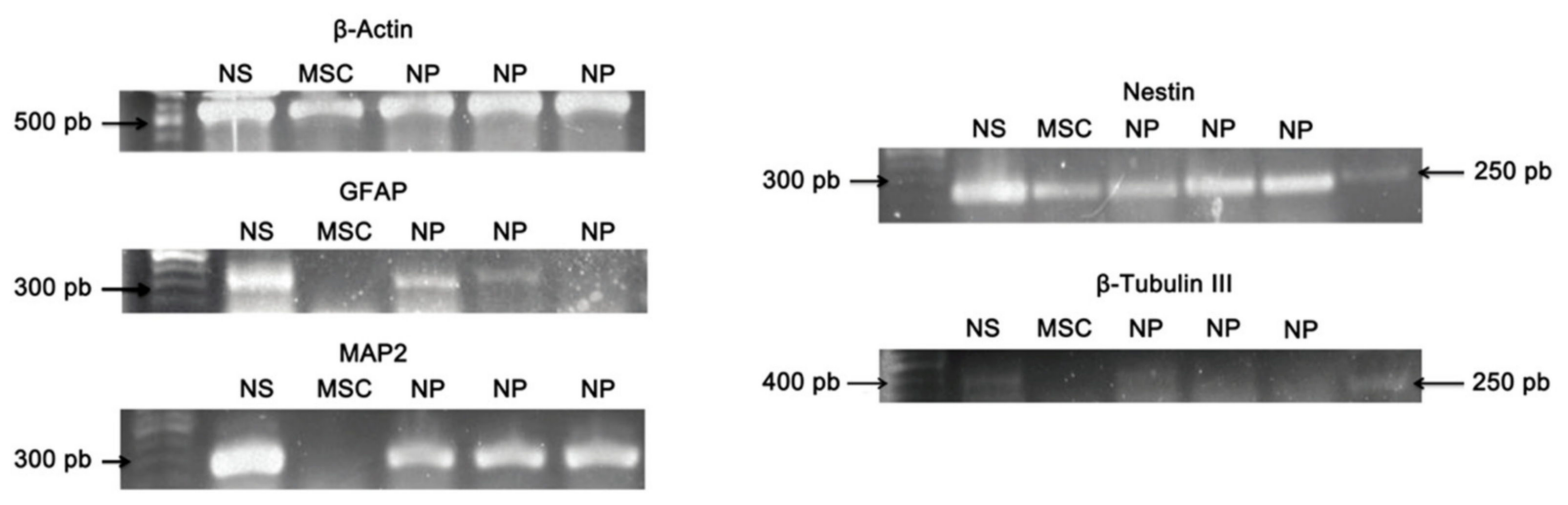
RT-PCR for markers of precursor neural cells. The ß-ACTIN gene was used as a technique control, and expression was observed for all samples. The GFAP gene was expressed in NS and NP samples. The *MAP2* gene was expressed in NS and NP’s gene. The NESTIN gene was expressed for all samples, and the ßIII-TUBULIN was observed in NS and NP samples. Abbreviations: not dissociated neurospheres: NS; MSC-WJ: MSC; neural precursor cells: NP.

In this study, the RT-PCR technique was used to evaluate the presence of molecular markers from neural precursor cells. We evaluated the expression of GFAP, MAP2, NESTIN, and ßIII-TUBULIN genes that are not dissociated neurospheres (NS), cultured neural precursor cells (NP), and MSC-WJ (MSC). Only mesenchymal cells from Wharton’s jelly were tested due to the higher cell yield in isolation and expansion. The ß-ACTIN gene was evaluated as a control gene expression. Cells from the neurospheres (NS) showed expression for all the neural genes tested, and the cultured neural precursor cells (NP) also expressed all genes, however, with weaker expressions for ßIII-TUBULIN, and GFAP, as demonstrated in Figure 5. The MSC-WJ (MSC), which was not cultured on the NFBX as membrane, presented expression only for the NESTIN gene. Thus, these results confirm the efficacy of the induction protocol to neural precursor cells using the NFBX.

### 3.7. Atomic Force Microscopy Analysis

**Figure 6:**
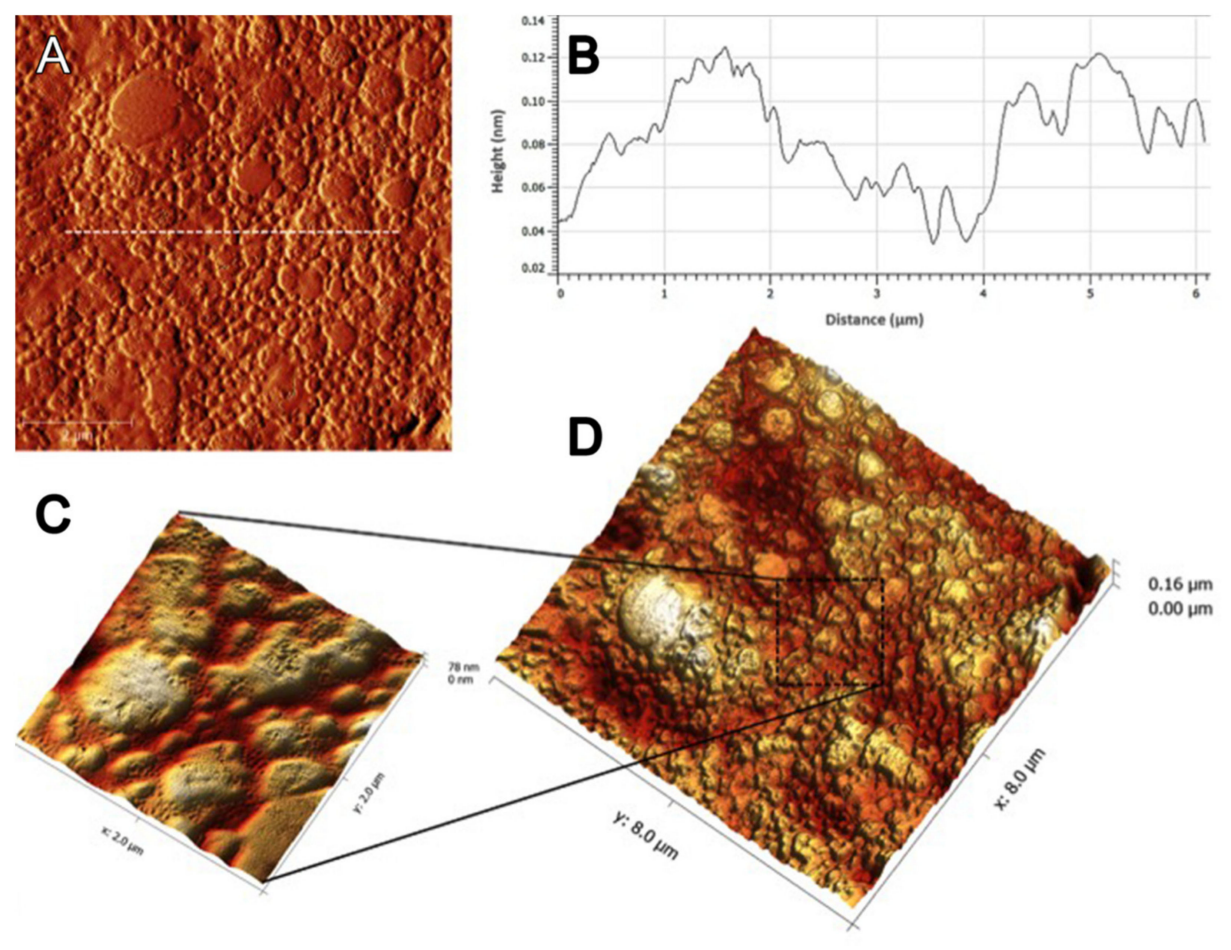
Atomic force microscopy images of the NFBX surface. (**A**) AFM height image; (**B**) Cross-section height profile (taken along the white line in the image (**A**) and (**C**,**D**) 3D AFM topographic images. Ra = 14.28 ± 3.28 nm.

Atomic Force demonstrated the roughness value, Ra = 14.28 ± 3.28 nm as shown in Figure 6.

### 3.8. Cholinergic Differentiation

**Figure 7:**
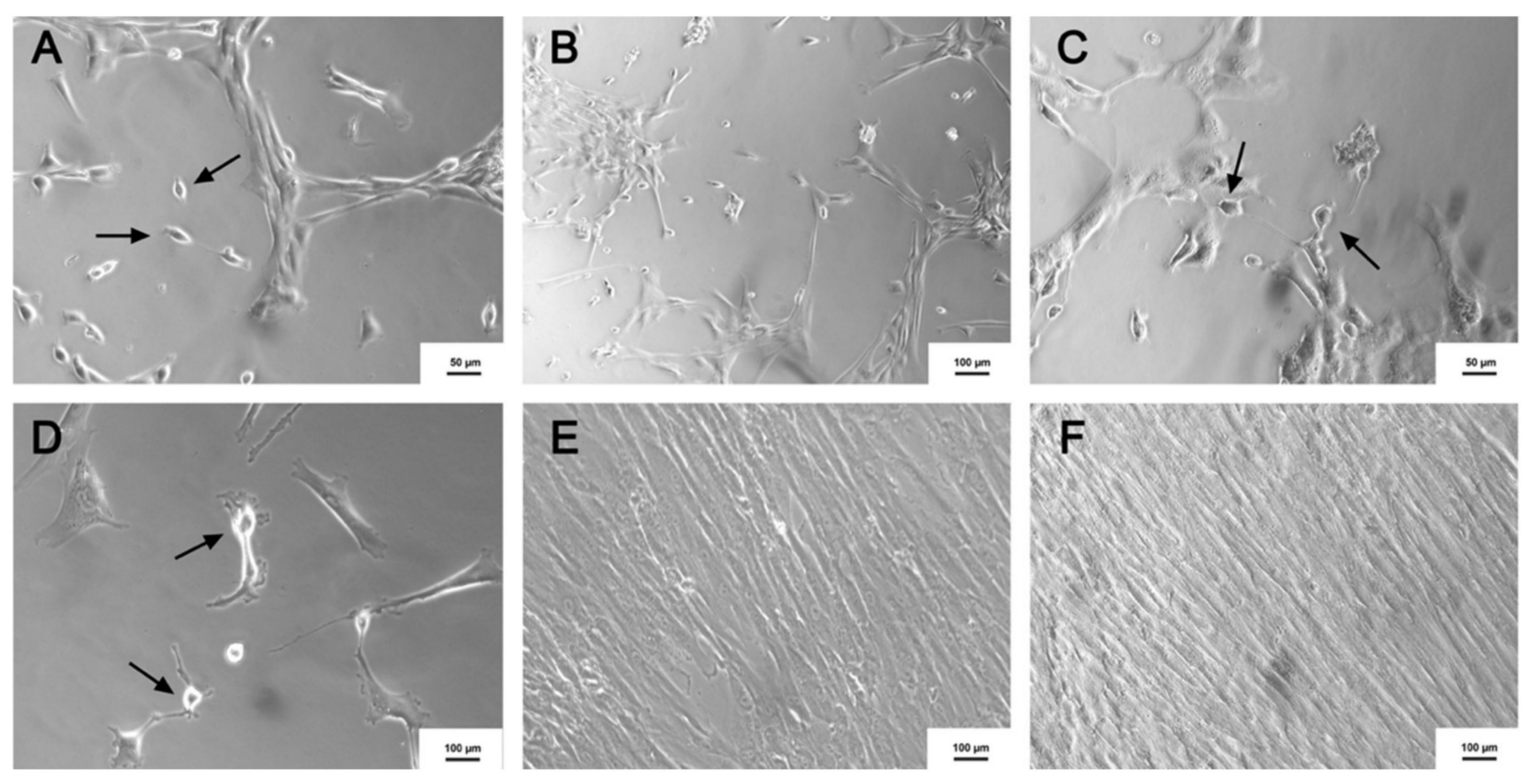
Cholinergic-like neurons differentiation. (**A**) 4th day after the beginning of the induction (×100); (**B**) 8th day after induction (×100); (**C**) 10th day after induction (×100); (**D**) 14th day after induction (×200); (**E**) Control (MSC) without the use of induction (×100); (**F**) Precursor without induction (×100). Black arrows represent the formation of the cholinergic-like neurons. (Image obtained using an inverted microscope x100, Axio Vert A1, Carl Zeiss, Oberkochen, Germany).

At the beginning of the cholinergic differentiation protocol, morphological differences were observed in some cells, which showed bipolar characteristics as demonstrated in Figure 7A. During the differentiation, a decrease was noticed in the number of MSC and an increase in the number of morphologically altered cells as identified in the cholinergic-like neurons, as shown in Figure 7B–D. Regarding this morphology, similar results were obtained by another study [19]. Controls that were performed without using induction factors did not present any morphological differences, as demonstrated in Figure 7E,F.

**Figure 8:**
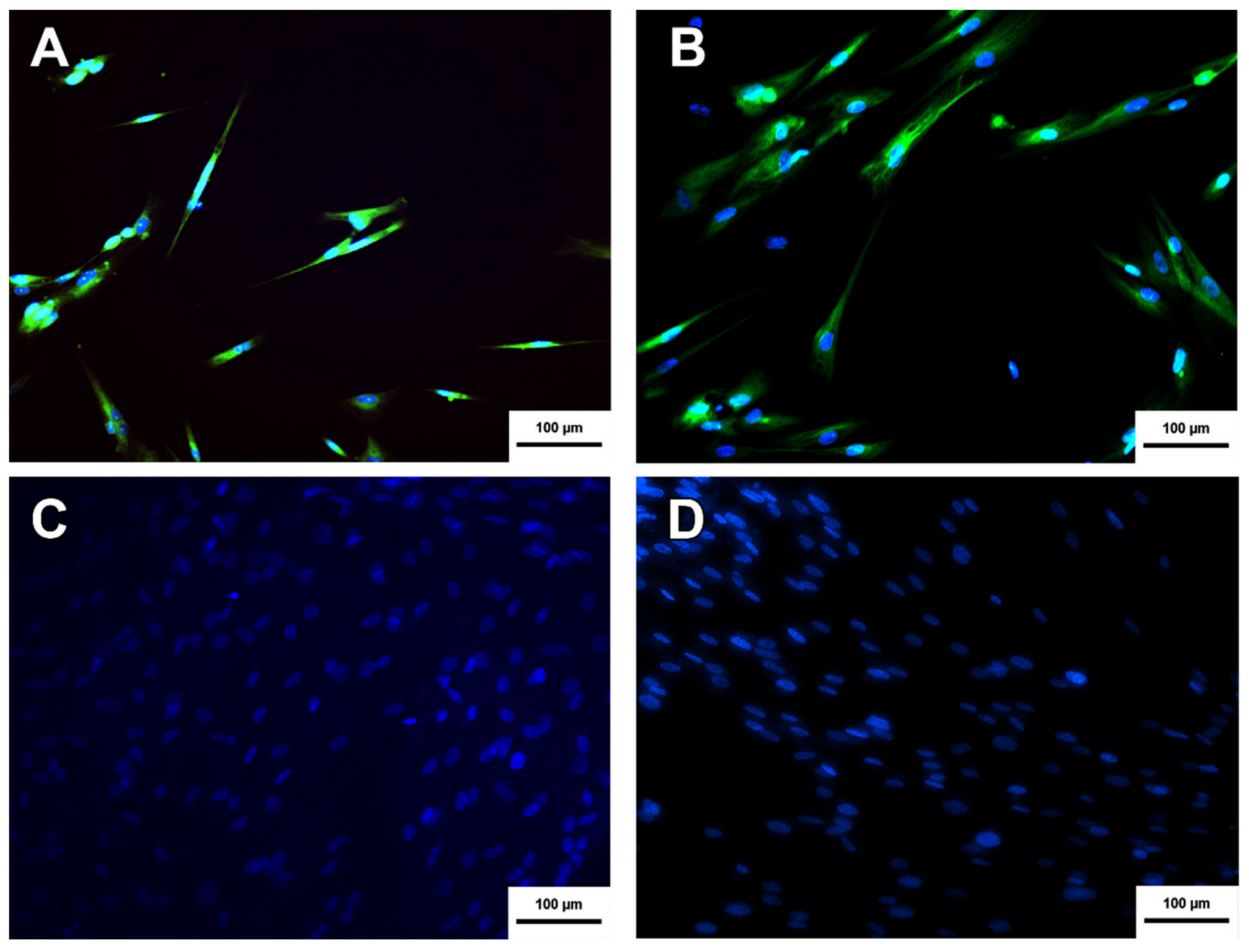
Cholinergic differentiation immunocytochemistry of NPCN+ cells derived from MSC-WJ. (**A**) MSC-WJ labeling using the anti-ChAT antibody (FITC). (**B**) MSC-WJ labeling using the anti-ßIII-TUBULIN antibody (FITC). (**C**,**D**) represent the negative controls (MSC-WJ not differentiated) for both markers anti-ChAT and anti-ßIII-TUBULIN, respectively Hoechst was used for labeling the nuclei (Image obtained using an inverted fluorescence microscope ×100, Axio Vert A1, Carl Zeiss, Oberkochen, Germany).

**Figure 9:**
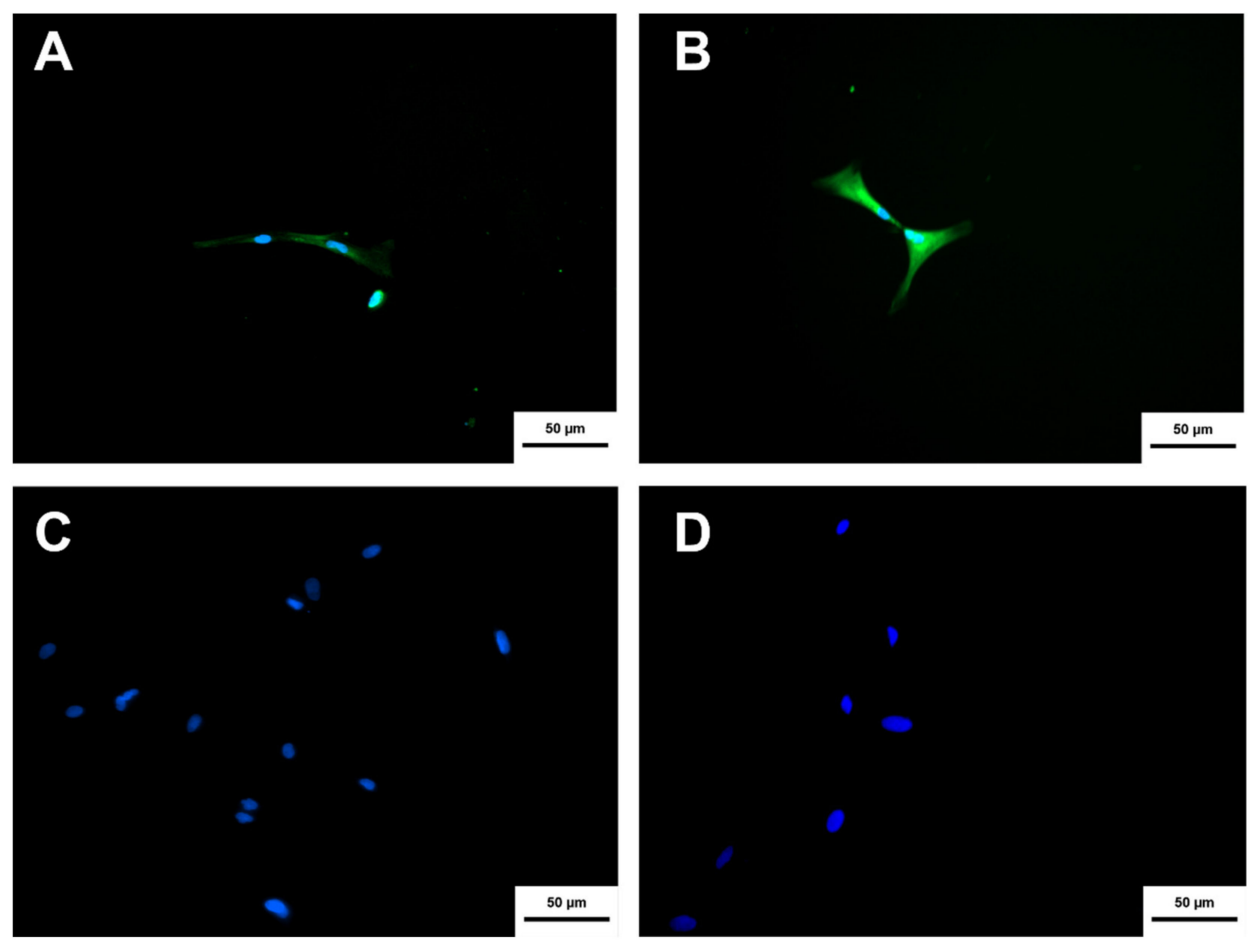
Cholinergic differentiation immunocytochemistry of NPCN + cells derived from MSC-UCB. (**A**) MSC-UCB labeling using the anti-ChAT antibody (FITC). (**B**) MSC-UCB labeling using the anti-ßIII-TUBULIN antibody (FITC). (**C**,**D**) represent the negative controls (MSC-UCB not differentiated) for both markers anti-ChAT and anti-ßIII-TUBULIN, respectively Hoechst was used for labeling the nuclei. (Inverted fluorescence microscope with increase of ×100, Axio Vert A1, Carl Zeiss, Oberkochen, Germany).

Neurospheres derived from MSC-WJ, and MSC-UCB presented different morphology when subjected to the cholinergic differentiation protocol. The differentiated cells were characterized by immunocytochemistry to verify the presence of two proteins, one that demonstrated the neural characteristic of the cell, ßIII-TUBULIN, and another that demonstrated the cholinergic property of the cell, the enzyme choline acetyltransferase. The differentiated cells from both sources (MSC-WJ and MSC-UCB) showed the expression of both proteins, as presented in Figure 8 and Figure 9. Negative controls were performed with neurospheres without differentiation induction, which showed the labeling for ßIII-TUBULIN, but did not show a label for the enzyme choline acetyltransferase.

## 4. Discussion

The umbilical cord is an essential cellular source, particularly of stem cells, mainly because it is a disposal material in hospitals and constitutes an important potential source for therapeutic applications. Both hematopoietic cells, used in transplants for the treatment of hematologic diseases, for example, and MSCs can be isolated from this material, making it a significant highlight in research in recent years [16].

In this study, the isolation of umbilical cord MSC from both Wharton jelly and umbilical cord blood was successfully performed considering adapting the techniques [8,12,13,14,20]. MSC-WJ isolated by employing the explant technique was satisfactory [12]. The isolation of MSC-UCB was performed by density gradient where fast cell adhesion could be observed, but the confluence took longer, 30 to 60 days on average, unlike another study that could observe confluent cells in 20 days culture [13,14].

According to the results described, MSC-UCB and MSC-WJ presented expression for the pre-established markers (CD73+, CD90+, CD105+, CD34- and CD45-). Also, the cells had the characteristic of adherence to the plastic substrate and fibroblast-like morphology. Cellular plasticity was demonstrated through chondrogenic, osteogenic, adipogenic, and neurogenic differentiation [4,13,21,22,23,24].

Authors who developed a cellular automata model concluded that cell death while resulting in a decrease in growth rate and final size of neurospheres, increases the differentiation potential of neural precursor cells, similar to that observed on a kinetic curve of growing cell culture [25]. The use of NFBX as a substrate for MSCs cultivation could provide cell interaction and related metabolism changes and, consequently, MSCs differentiation. Future studies need to give a proteomic and genomic explanation for how MSC can form neurospheres and differentiate into neural precursors cells when seeded in NFBX. The authors discussed the relationship between this membrane and cell fate and pointed out that geometric conditions interacting with and cellular cytoskeletal protein associated with microdomains of plasma membrane lipid rafts seem to regulate unknown signals [26]. In the membrane used, it was observed that, due to the atomic force (Figure 6), the roughness value could be responsible for the low adhesion and selection of the nestin-positive mesenchymal stem cells for the differentiation in their cellular fate by aggregating the neural precursor cells in neurospheres. The culture of neurospheres is widely described in the literature because it is a crucial way to expand the production of NSCs, and several neural differentiation protocols are reported [1].

Petersen et al. (2018) demonstrated that stem cell subpopulation in rat adults from adipose tissue was directly expanded in NSCs. One of their changes was to reduce the costs and time required to derive and expand NSCs. The protocol to form neurospheres was composed of neurobasal medium supplemented with Glutamax; B-27; N2; bFGF, and EGF. This protocol was able to form neurospheres with characteristics of neural stem cells, and these cells expressed NESTIN, SOX2, TUJI, and GFAP proteins. The amount described is much higher than the used in this study, which was only 2 × 10^4^ cells/mL. Although the sources of formation were different, the present study does not use growth factors, and the number of cells necessary to produce neurospheres was lower [27].

It is important to emphasize that this study does not aim to demonstrate the mechanisms of this natural membrane that determined the formation of neurospheres. When considering neurospheres obtained from umbilical cord-derived MSCs, most authors used growth factors or gene transfection to induce neural precursor cells [8]. Some protocols used neurobasal medium and growth factors such as EGF, N2, bFGF, and B27 to induce neurospheres production [8,28]. In contrast, the present study used conventional media and no supplementation in the culture medium to produce neurospheres from undifferentiated MSCs, regardless of MSC-WJ or MSC-UCB, only the NFBX was coating the culture flasks before hMSCs plating was conducted. The NFBX method made this study more economical, practical, and without the drawbacks of inductions [27].

Also, this method of cultivation provides a cleaner environment for interferers with considering future therapeutic applications, following Good Manufacturing Practice (GMP). The neurospheres obtained from seeded MSC-WJ and MSC-UCB in NFBX were characterized by immunocytochemistry for the nestin protein, and the presence of this protein was demonstrated by immunofluorescence [1,4,8]. Nestin is an intermediate filament protein present mainly in neural precursor cells and other cell types such as NSCs and in some adult neurons, mainly in regeneration areas, thus indicating a marker that demonstrated commissioned to neurons and proliferation capacity [18,29]. Besides these facts, SEM results shown that NFBX provides a cell organization originating from the neurospheres. The monolayer-forming by MSC-WJ organize themselves through anchor cells, and cells migrate to the formation of neurospheres.

The same protocol to produce neurospheres derived from MSC-WJ was used to produce neurospheres derived from the MSC-UCB; however, the time required to form neurospheres from the last source was longer. Some authors explain that this difficulty is due to the few precursors of MSCs circulating in the umbilical cord blood, most of which reside in the adult organism [30].

Differentiated neural precursor cells in cholinergic-like neurons s could be observed in all sources of MSCs. These cholinergic-like neurons express a cholinergic phenotype. The immunophenotypic labeling for both the choline acetyltransferase protein produced by cholinergic neurons and the ßIII-TUBULIN protein, which is a protein involved in forming microtubules in neurogenesis also was observed in the two sources of neurospheres. The growth factors used in this protocol were seen to be useful for the differentiation of neural precursor cells to cholinergic phenotype cells, corroborating results obtained by other authors [19]. Those authors stated that the medium used for cholinergic differentiation, which is characterized by the gradual decrease of EGF and bFGF and the gradual increase of NGF, acted as the main factor of cholinergic differentiation and allowed the survival of the cell trying to resemble the natural conditions of a living organism. [19].

The genes used to characterize the neurospheres are extensively described in the literature in various MSC sources; *NESTIN* as a neural precursor cell marker, *MAP2* and *GFAP* are considered glial markers of mature neurons, and *ßIII-TUBULIN* is specific for neuron cytoskeleton [8,31,32,33,34,35]. The *NESTIN* gene expression in MSC-WJ, which was not cultured on the NFBX, means that these cells did not induce the formation of neural precursor cells, confirming results obtained by another study, in that MSC-WJ presented expression of the nestin gene in the undifferentiated form [32]. Such expression increased when cells were induced to form neurospheres [32,36]. According to this study, MSC-WJ demonstrated higher efficiency in neural precursor differentiation when compared to other sources described, such as adipose tissue and bone marrow. Considering the expression of *NESTIN*, *MAP2*, *GFAP*, and *ßIII-TUBULIN* genes on neurospheres and on cultured neural precursors cells derived from MSC-WJ, the results obtained in this study followed the gene pathways found by another study [1].

Questions remain to be investigated in order to understand the development of these neurospheres from the MSCs’ contact with this membrane: first, to provide more details of the morphologies and cell interactions; second, how the precise gene expression patterns from a heterogeneous population of MSCs could select precisely the cells that would be committed to being precursors of neurons; and, third, to investigate how cells communicate and make decisions during spheroid formation and how shape and morphology emerge by mechanic-physicochemical processes to determine their geometry.

This study is limited to demonstrating the cholinergic phenotype neurons with three proteins: NESTIN, ChAT, and ßIII-TUBULIN, although there is a risk of a dichotomy between mRNA consequent gene expression and protein levels. The scarcity of RNA materials for analysis was explained by the reduced cell number when submitted to differentiation. Further studies are needed to understand the mechanisms of the functionalities of NFBX to promote the aggregated hMSCs in neurospheres. Those facts are probably due to epigenetic factors not known yet.

## 5. Conclusions

This protocol demonstrates obtaining the neurospheres without growth factors or gene transfection, ideal for translation proceedings, only seeding the undifferentiated MSCs on the natural biopolymer membrane coating in the substrate.

This study suggests a potential use of the human neural precursor cells obtained from this protocol and followed by cholinergic differentiation for cholinergic-like neurons for analysis in preclinical models of Alzheimer’s Disease (in press) before translation.

This type of cholinergic-like neurons could promise another safe way to repair the loss of cholinergic neurons.

## Data Availability

Not applicable.

## Supplementary Materials

The following are available online at https://www.mdpi.com/article/10.3390/membranes11080598/s1, Figure S1: Flow Cytometry Histograms and table analysis. Table S1. Cholinergic differentiation protocol in accordance Adib et al., 2015.
