# Supplementary material for: Human Mesenchymal Stem Cells Seeded on the Natural Membrane to Neurospheres for Cholinergic-like Neurons
Source: Membranes (Basel). 2021 Aug 7;11(8):598. doi: 10.3390/membranes11080598 (PMC8400270; doi:10.3390/membranes11080598)
Supplement: Supplementary file 1 [file membranes-11-00598-s001.zip › membranes-1314593-supplementary.pdf]

membranes

Supplementary Material

Human Mesenchymal Stem Cells Seeded on the Natural Membrane to Neurospheres for Cholinergic-Like Neurons

Priscila Elias Ferreira Stricker [1,†], Daiany de Souza Dobuchak [1,†], Ana Carolina Irioda [1], Bassam Felipe Mogharbel [1], Celia Regina Cavichiolo Franco [2], José Roberto de Souza Almeida Leite [3], Alyne Rodrigues de Araújo [4], Felipe Azevedo Borges [5], Rondinelli Donizetti Herculano [5], Carlos Frederico de Oliveira Graeff [6], Juan Carlos Chachques [7] and Katherine Athayde Teixeira de Carvalho [1,*]

1 Advanced Therapy and Cellular Biotechnology in Regenerative Medicine Department, Child and Adolescent Health Research and Pequeno Príncipe Faculties, Pelé Pequeno Príncipe Institute, 80240-020 Curitiba, Brazil; priscilaeferreira@gmail.com (P.E.F.S.); daianys.bio@gmail.com (D. S. D); anairioda@gmail.com (A.C.I.); bassamfm@gmail.com (B.F.M.)
2 Cell Biology Department, Federal University of Paraná, 81530-000 Curitiba, Brazil; crcfranc@terra.com.br
3 Research Center in Applied Morphology and Immunology, NuPMIA, Faculty of Medicine, University of Brasília, 70910-900, Brasília, Brazil; jrsaleite@gmail.com
4 Biodiversity and Biotechnology Research, Parnaíba Delta Federal University, 64202-020 Parnaíba, Brazil; alyne_biomed@hotmail.com (A.R.A)
5 Faculty of Pharmaceutics Sciences, São Paulo State University (UNESP), 14800-903 Araraquara, Brazil; felipeazevedoborges@hotmail.com (F.A.B.); rond.donizetti@gmail.com (R.D.H.)
6 Physics Department, São Paulo State University (UNESP), 17033-360 Bauru, Brazil; carlos.graeff@unesp.br (C.F.O.G)
7 Pompidou Hospital, Cardiovascular Division. Laboratory Biosurgical Research, University of Paris 75015 Paris, France; j.chachques-ext@aphp.fr
* Correspondence: katherinecarv@gmail.com; Tel.: +55-41-3310-1035.
† These authors contributed equally to this work.

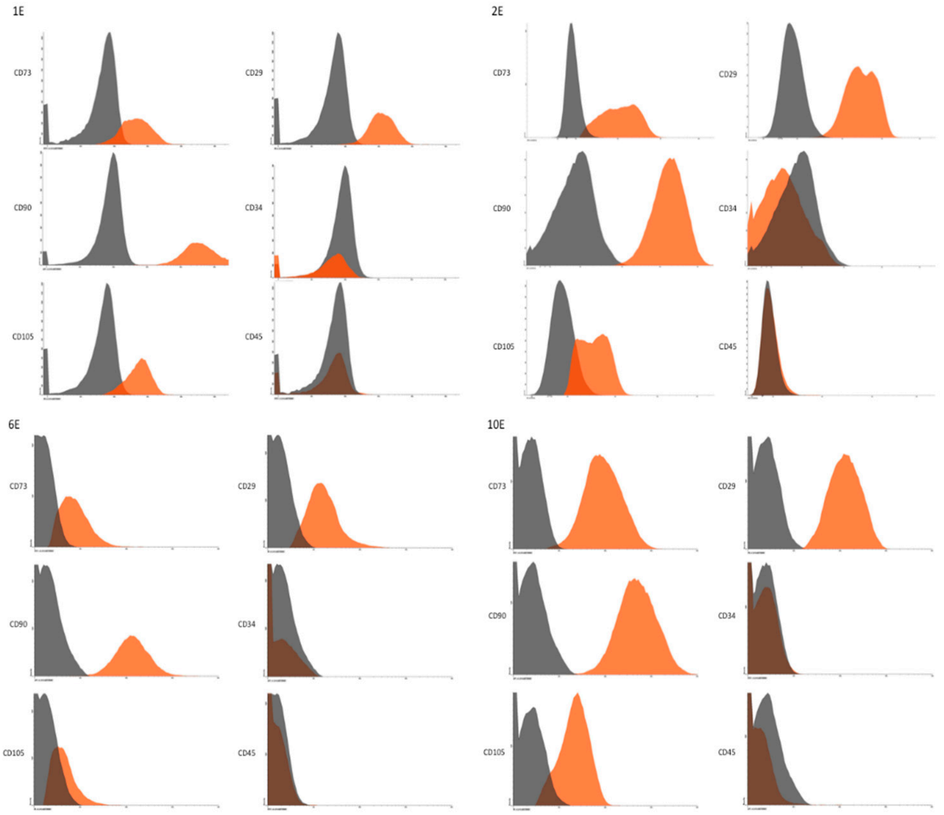

Surface marker expression of MSC-WJ. Note: SD – standard deviation. Referring to samples 1E, 2E, 6E and 10E.

Marker	CD73/CD105/CD90 (+)	CD73/CD29 (+)	CD34/CD45 (-)	7AAD (+)
1E	87.72	94.28	99.90	7.45
2E	91.75	89.61	99.97	15.67
6E	91.83	--	99.67	34.54
10E	93.76	--	99.99	12.95
Mean	91.27%	91.95%	99.88%	17.65%
SD	0.03	0.03	0.00	0.12

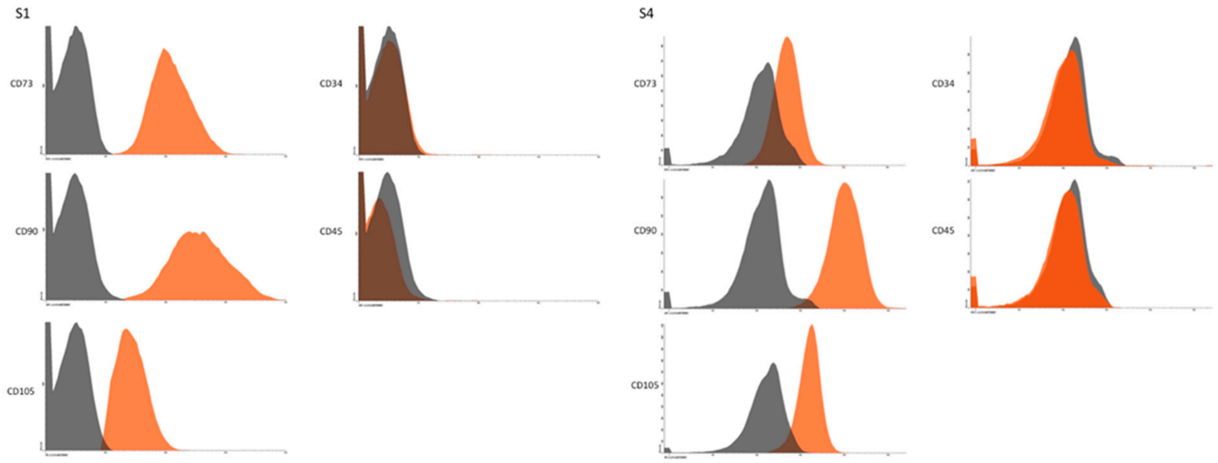

Surface markers expression of MSC-- - -UCB. Note: SD – standard deviation. Referring to samples S1 and S4.

Marker	CD73/CD105/CD90 (+)	CD34/CD45 (-)	7AAD (+)
S1	90.26	99.75	11.47
S4	92.33	99.95	6.49
Mean	91.30%	99.85%	10.53%
SD	1.46	0.13	6.01

Figure S1. Flow Cytometry Histograms and table analysis. (A) Histograms for the samples 1E, 2E, 6E, and 10E from MSC-WJ, and the respective table showing the statistic values. (B) Histograms for the samples S1 and S4 from MSC-UCB, and the respective table showing the statistic values. The gray peaks represent the isotype controls determining the area in which the marker is negative. The orange peaks correspond to the sample marking with its respective immunostaining marker, which is positive when the right of the gray peak and negative when on top or the left of the gray peak. Flow Cytometer was performed in FACS Calibur; Becton Dickinson, USA, and the analysis were conducted using Infinicyt Flow Cytometry Version 1.6.0 software.

Table S1. Cholinergic differentiation protocol in accordance Adib et al.,2015. A different concentration of growth factors and B27 per mL of medium was required throughout the 11 days of the protocol.

Day	NGF ng/mL	EGF ng/mL	bFGF ng/mL	B27 %
**0**	0	10	10	1
**1**	10	9	9	0.9
**2**	20	8	8	0.8
**3**	30	7	7	0.7
**4**	40	6	6	0.6
**5**	50	5	5	0.5
**6**	60	4	4	0.4
**7**	70	3	3	0.3
**8**	80	2	2	0.2
**9**	90	1	1	0.1
**10**	100	0	0	0
